# Pax8 as a useful adjunct marker to differentiate pancreatic serous cystadenoma from clear cell renal cell carcinoma in both cytologic and surgical specimens

**DOI:** 10.1186/s13000-023-01339-3

**Published:** 2023-04-25

**Authors:** Zhikai Chi, Jing Xu, Dipti M. Karamchandani, Lan Peng

**Affiliations:** Department of Pathology, The University of Texas Southwestern Medical Center, 5323 Harry Hines Blvd, NPL02-950, Dallas, TX 75390 USA

**Keywords:** Pancreatic serous cystadenoma, Clear cell renal cell carcinoma, Pax8, Differential diagnosis, Immunohistochemistry

## Abstract

**Background:**

Histomorphological differentiation between pancreatic serous cystadenoma (SCA) and clear cell renal cell carcinoma (RCC) can be challenging. We aimed to study Paired box 8 protein (Pax8) expression profile in cytologic and surgical specimens with pancreatic SCA to assess its utility as a differentiating marker from clear cell RCC.

**Methods:**

We characterized Pax8 immunohistochemistry in 33 patients with pancreatic SCA (23 surgical resections and 10 cytology specimens). Nine cytology specimens from metastatic clear cell RCC involving pancreas were used as control tissue. Electronic medical records were reviewed to retrieve clinical information.

**Results:**

All 10 pancreatic SCA cytology specimens, and 16 of 23 pancreatic SCA surgical resections showed absent Pax8 immunostaining, while the remaining 7 surgical resection specimens showed 1%-2% immunoreactivities. Islet and lymphoid cells adjacent to the pancreatic SCA expressed Pax8. In contrast, the proportion of Pax8 immunoreactivity ranged from 50 to 90% (average of 76%) in nine cases of metastatic clear cell RCC involving pancreas. Using a 5% immunoreactivity cutoff, all cases of pancreatic SCA are interpreted as negative for Pax8 immunostains while all cases of metastatic clear cell RCC involving pancreas are interpreted as positive for Pax8 immunostains.

**Conclusions:**

These results suggest that Pax8 immunohistochemistry staining can be a useful adjunct marker to differentiate pancreatic SCA from clear cell RCC in clinical practice. To the best of our knowledge, this is the first large-scale study of Pax8 immunostaining on surgical and cytology specimens with pancreatic SCA.

## Key messages

- Pax8 expression in pancreatic serous cystadenoma is rare (< 5% immunoreactivity).
- Using a 5% immunoreactivity cutoff, all cases of pancreatic SCA are interpreted as negative for Pax8 immunostains while all cases of metastatic clear cell RCC involving pancreas are interpreted as positive for Pax8 immunostains.
- Adjacent islet and lymphoid cells expressing Pax8 can be potential diagnostic pitfalls

## Background

Pancreatic serous cystadenoma (SCA) is a benign, yet important, epithelial neoplasm that is largely composed of small uniform cuboidal glycogen-rich cells that often form cysts containing serous fluid [1, 2]. Imaging studies, such as computed tomography (CT) or endoscopic ultrasonography (EUS), largely facilitate the diagnosis of SCA [2, 3]. However, cytohistological evaluations remain the gold standard for a definitive diagnosis [2, 4–6].

In clinical practice, metastatic clear cell renal cell carcinoma (RCC) comes in the differential diagnosis for pancreatic SCA, especially in patients with von Hippel-Lindau (VHL) disease who have a propensity to develop both diseases [7, 8]. Morphologically, pancreatic SCA and clear cell RCC share some similar characteristics with glycogen-rich cells, abundant clear cytoplasm, distinct cytoplasmic borders, and rich vascularity [1, 9]. In clear cell RCC, grade 1 or 2 classification based on the World Health Organization (WHO) /International Society of Urologic Pathology (ISUP), tumor nucleoli can be inconspicuous or absent, which mimics the nucleoli of pancreatic SCA [9]. Furthermore, cytology specimens are usually small or paucicellular, containing limited tissue for pathological analyses. Given the significant management and prognostic implications involved in differentiating pancreatic SCA (an essentially benign neoplasm) and clear cell RCC (a carcinoma), pathologists often rely on ancillary immunohistochemical staining for a definitive diagnosis. Paired box 8 protein (Pax8), carbonic anhydrase 9 (CAIX), and CD10 are the most frequently used positive immunochemical markers in context for clear cell RCC [8, 10–15]. Unfortunately, both CAIX and CD10 are expressed in pancreatic SCA and are, thus, not particularly useful to help distinguish between these two entities [16, 17]. In contrast, Pax8 was recently shown to be negative in pancreatic SCA in a small study involving five patients with VHL disease [8]. However, Pax8 expression profile studies have not been conducted in larger pancreatic SCA case series.

Based on this limited evidence pointing to a lack of Pax8 expression data in pancreatic SCA, we aimed to characterize the Pax8 expression profile in a larger series of pancreatic SCA and study its significance with respect to clinical parameters and histological features. To the best of our knowledge, this is the first large study looking at the Pax8 expression profile in both surgical resection and cytologic specimens of pancreatic SCA.

## Methods

### Study design and histomorphological analysis

This study was approved by the Institutional Review Boards of the University of Texas Southwestern Medical Center and Parkland Health and Hospital System. Thirty-three cases registered in the surgical pathology database that met the diagnostic criteria for pancreatic SCA were analyzed. Nine metastatic clear cell RCC involving pancreas were analyzed as control cases. Hematoxylin and eosin slide from cell blocks (endoscopic ultrasound-guided fine-needle aspiration cytology cases), or tissue sections (surgical resection cases) and immunohistochemistry slides were reviewed by two pathologists (ZC and LP) to confirm the original pathological diagnosis in each case. The diagnosis of serous cystadenoma are largely dependent on histopathologic features including cystic spaces lined by a single layer of cuboidal to low columnar bland appearing clear cells containing generous amount of glycogen rich clear cytoplasm; the dividing septae between the spaces usually show an extensive network of subepithelial vessels; the SCA nuclei are generally bland, small, round, and uniform with inconspicuous nucleoli. Although scattered cells can show degenerative atypia, mitoses are typically absent [18]. Electronic medical records were reviewed to collect data on patient age, sex, radiological findings, clinical chemistry results, and clinical follow-up information.

### Immunohistochemical analysis

The primary antibodies used were anti-human Pax8 (MRQ-50, prediluted, Cell Marque) and anti-human inhibin (alpha, R1, prediluted, Cell Marque). Immunohistochemical staining for Pax8, and Inhibin was performed on formalin-fixed, paraffin-embedded, 4-µm tissue sections using a Ventana Benchmark automated immunostainer (Ventana, Tucson, AZ, USA). Tissue sections were deparaffinized, and localization of the antigen–antibody complex was achieved using the OptiView DAB detection kit. Antigen retrieval was performed using heat-induced epitope retrieval for 32 min for Pax8, or 64 min for inhibin, in cell conditioning 1 buffer.

Pax8 immunoreactivity was interpreted using nuclear staining alone, whereas inhibin immunoreactivity was interpreted using cytoplasmic staining [2, 10, 11, 19]. The percentage of Pax8- or inhibin-immunoreactive tumor cells was scored as a proportion. Representative fields were selected and imaged at 400 × magnification using the same microscope. A 5% immunoreactivity cutoff was used to classify a case as positive.

### Statistical and survival analysis

Categorical data were analyzed using Pearson’s chi-square test or Fisher’s exact test if the number of cases in a category was less than ten. Continuous numerical data were analyzed using Student’s t-test. All statistical analyses were based on a two-sided significance level of *p* < 0.05. The IBM SPSS Statistics 28 software was used for all analyses.

## Results

### Demographics and clinicopathological features

The clinicopathological features of the 33 patients included in this study are summarized in Table 1. The average age at diagnosis was 63 years and 70% of the patients were females. Ten endoscopic ultrasound-guided fine-needle aspiration (EUS-FNA) cytology specimens and twenty-three surgical resection specimens were analyzed. Notably, six patients had a history of VHL syndrome. Among 11 patients with a history of detectable cyst fluid carcinoembryonic antigen (CEA), 2 patients showed elevated levels (> = 5 ng/mL), whereas the rest were normal [20]. Among the ten EUS-FNA cytology cases, clinical follow-up information was available for eight patients, with an average follow-up period of 35 months (range 4–76 months). At the time of last follow-up, six patients remained stable and asymptomatic, while two patients had succumbed to the other disease(s).

**Table 1:** Clinicopathological features of 33 patients with pancreatic serous cystadenoma

**Clinicopathological features**	Variables	n (%)
**Age (median, range)**	63 years (23–87)	
**Gender**	Male	10 (30%)
	Female	23 (70%)
**Specimen type**	Surgical resection	23 (70%)
	EUS cytology	10 (30%)
**Location**	Head	8 (24%)
	Body	16 (48%)
	Distal	8 (24%)
	Diffuse	1 (3%)
**Cyst size (median, range)**	2.6 cm (0.3–7.5)	
**Cyst fluid CEA level (*n* = 11)**	Normal (< 5 ng/mL)	9 (82%)
	Elevated (> = 5 ng/mL)	2 (18%)
**Cyst types**	Microcystic	26 (79%)
	Macrocytic/oligocystic	7 (21%)
**von Hippel-Lindau syndrome**	Yes	6 (18%)
	No	27 (82%)
Patient follow-up for EUS cytology cases (*n* = 10)	No follow-up available	2 (20%)
	Died of other disease(s)	2 (20%)
	Stable and asymptomatic	6 (60%)

### Inhibin and Pax8 immunoreactivity in pancreatic SCA

**Fig. 1:**
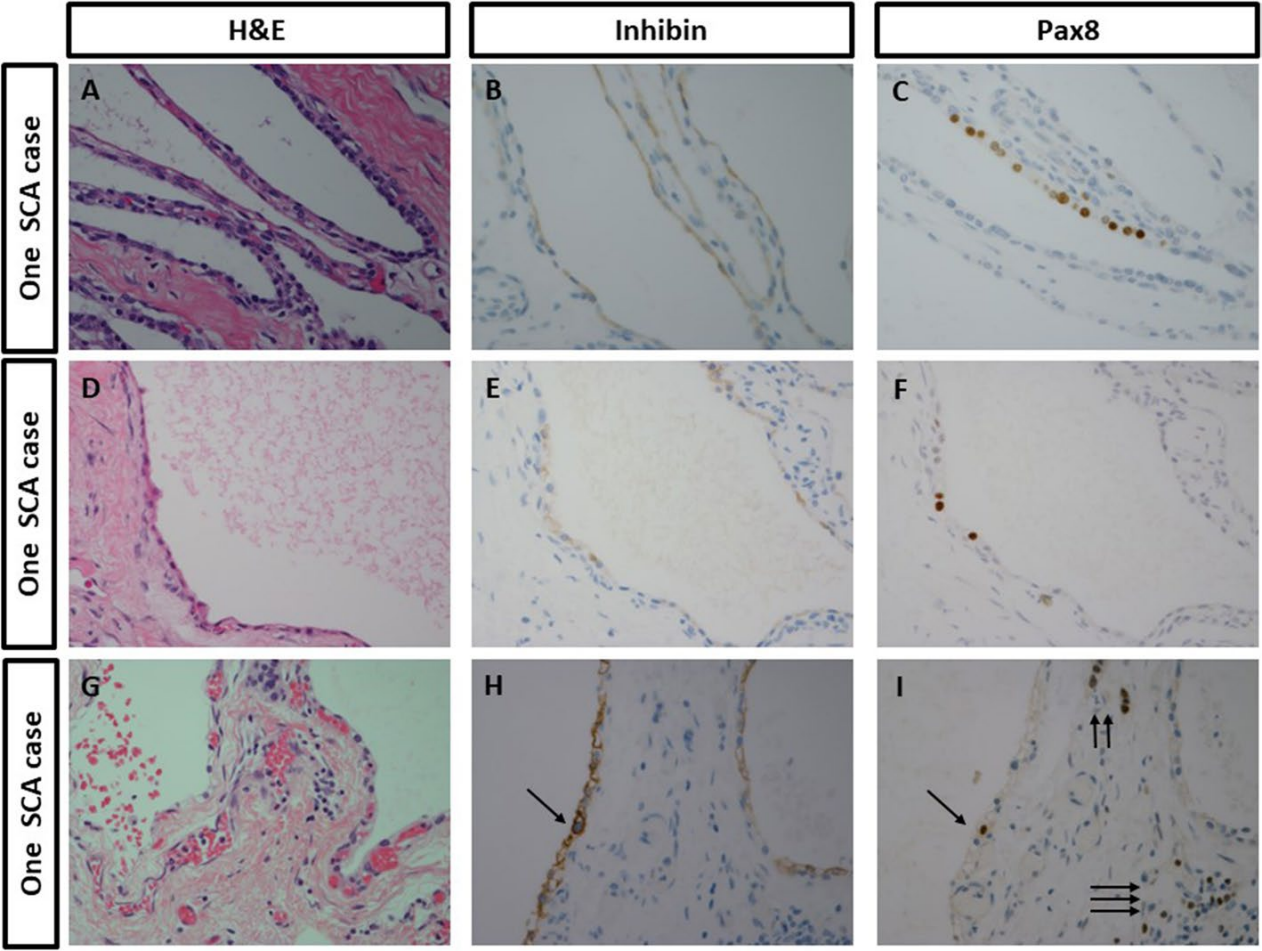
Representative images from pancreatic serous cystadenoma (SCA) surgical resection cases. **A**–**C** One pancreatic SCA surgical resection case. **D**–**F** One pancreatic surgical resection SCA case. **G**–**I** One pancreatic SCA surgical resection case. **A**, **D**, and **G** Hematoxylin and eosin (H&E) staining. **B**, **E**, and **H** Inhibin. **C**, **F**, and **I** Pax8. **H** and **I** Single arrow: one pancreatic SCA cell; **I** double arrows: islet cells; triple arrows: lymphoid cells. Magnification = 400 × (**A**–**I**)

**Fig. 2:**
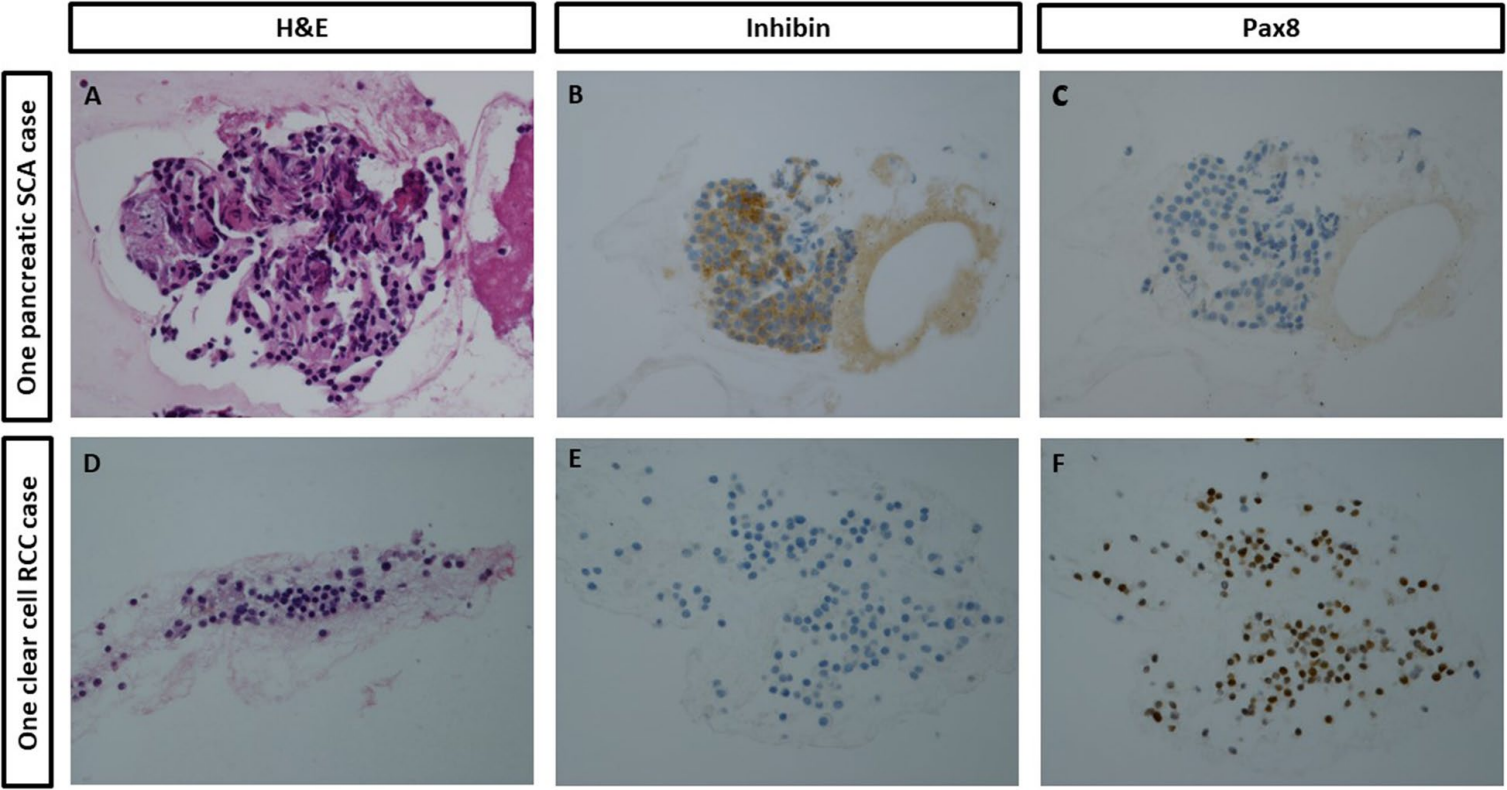
Representative images from one pancreatic serous cystadenoma (SCA) cytology case and one cytology case of metastatic clear cell renal cell carcinoma (RCC) involving pancreas. **A**–**C** One pancreatic SCA cytology case. **D**–**F** One cytology case of metastatic clear cell RCC involving pancreas. **A** and **D** Hematoxylin and eosin (H&E) staining. **B** and **E** Inhibin. **C** and **F** Pax8. Magnification = 400 × (**A**–**F**)

**Fig. 3:**
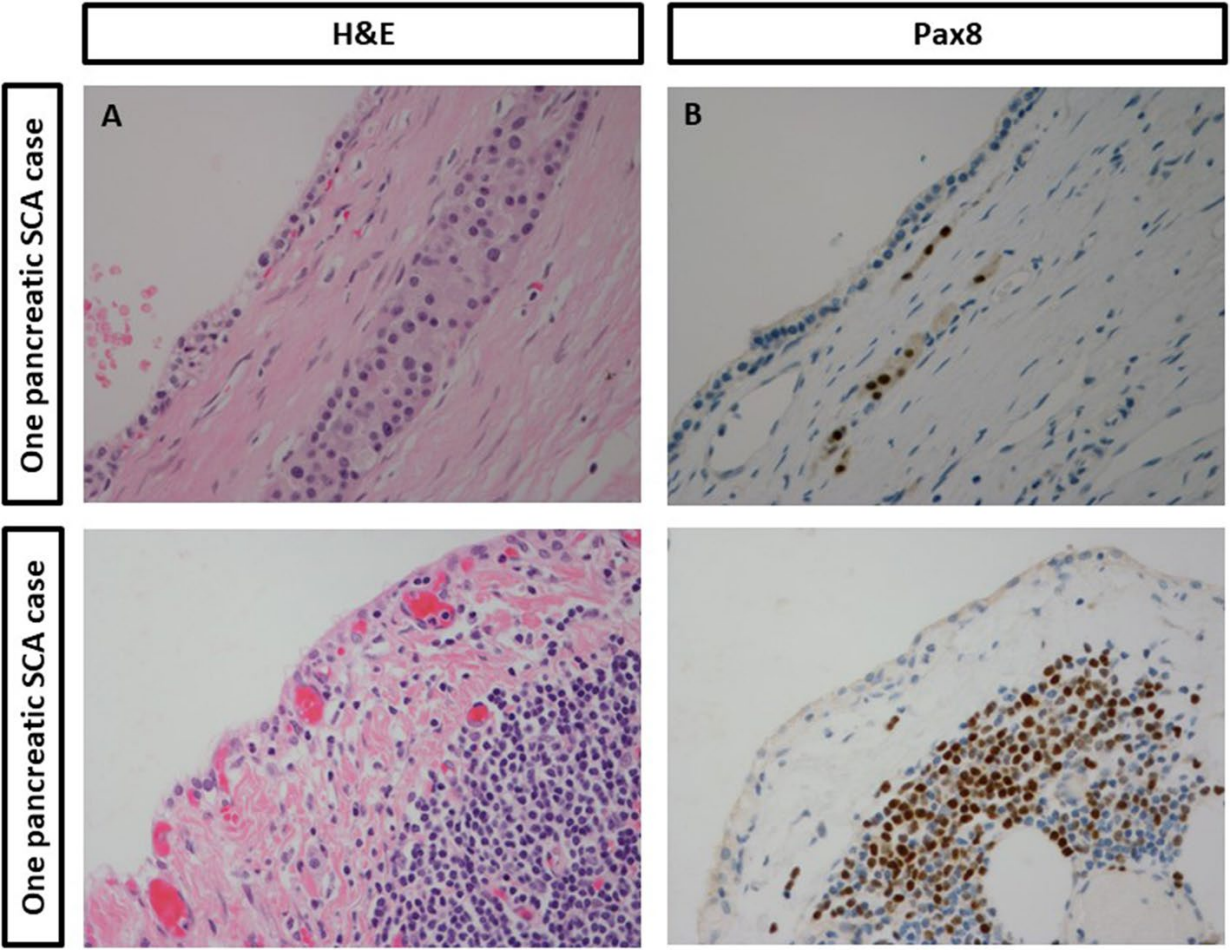
Representative images from pancreatic serous cystadenoma (SCA) surgical resection cases. **A**, **B** One pancreatic SCA surgical resection case highlighting islet cells. **C**, **D** One pancreatic surgical resection SCA case highlighting lymphoid cells. **A** and **C** Hematoxylin and eosin (H&E) staining. **B** and **D** Pax8. Magnification = 400 × (**A**–**D**)

Five out of 23 (22%) pancreatic SCA resection specimens and 1 out of 10 pancreatic SCA cytology specimens showed no inhibin immunoreactivity (Figs. 1 and 2). Details of the proportions of inhibin immunoreactivity are shown in Table 2. Sixteen of 23 (70%) pancreatic SCA resection specimens and all 10 pancreatic SCA cytology specimens showed no (0%) Pax8 immunoreactivity (Figs. 1 and 2, and Table 2). Among the remaining seven pancreatic SCA resection specimens, six showed 1% immunoreactivity, and one showed 2% immunoreactivity. Furthermore, this low (1–2%) Pax8 expression in pancreatic SCA was not associated with patient age, sex, location, cyst fluid CEA levels, cyst type (microcystic versus macrocytic), or history of VHL syndrome (Table 3). Notably, islet and lymphoid cells adjacent to the pancreatic SCA were positive for Pax8 (Figs. 1 and 3).

**Table 2:** Inhibin and Pax8 immunoreactivity in pancreatic serous cystadenoma and metastatic clear cell renal cell carcinoma involving pancreas

**Inhibin and Pax8 immunoreactivity in surgical specimen of pancreatic serous cystadenoma**					
** Inhibin proportion mean (%)**	39 (*n* = 23, range 0–90)				
** Inhibin proportion (%)**	0	1–5	6–25	26–50	> 50
	5 (22%)	0	5 (22%)	6 (26%)	7 (30%)
** Pax8 proportion mean (%)**	0.4 (*n* = 23, range 0–2)				
** Pax8 proportion (%)**	0	1–5	6–25	26–50	> 50
	16 (70%)	7 (30%)	0	0	0
**Inhibin and Pax8 immunoreactivity in cytology specimen of pancreatic serous cystadenoma**					
**Inhibin proportion mean (%)**	57 (*n* = 10, range 0–90)				
**Inhibin proportion (%)**	0	1–5	6–25	26–50	> 50
	1 (10%)	0	0	5 (50%)	4 (40%)
**Pax8 proportion mean (%)**	0 (*n* = 10, range 0–0)				
**Pax8 proportion (%)**	0	1–5	6–25	26–50	> 50
	10 (100%)	0	0	0	0
**Pax8 immunoreactivity in cytology specimen of metastatic clear cell renal cell carcinoma involving pancreas**					
**Pax8 proportion mean (%)**	76 (*n* = 9, range 50–90)				
**Pax8 proportion (%)**	0	1–5	6–25	26–50	> 50
	0	0	0	1 (11%)	8 (89%)

**Table 3:** Clinicopathological features of 33 patients with pancreatic serous cystadenoma with or without Pax8 expression

**Features**	Variables	No Pax8 expression (number, %)	Pax8 expression (number, %)	*P* value
**Age (mean, range)**	63 years (23–87)	64 years (23–87)	53 years (43–66)	0.07
**Gender**	Male	8 (80%)	2 (20%)	0.91
	Female	18 (78%)	5 (22%)	
**Location**	Head	7 (88%)	1 (13%)	0.84
	Body	12 (75%)	4 (20%)	
	Distal	6 (75%)	2 (25%)	
	Diffuse	1 (100%)	0	
**Cyst fluid CEA level (*n* = 11)**	Normal (< 5 ng/mL)	8 (89%)	1 (11%)	0.20
	Elevated (> = 5 ng/mL)	1 (50%)	1 (50%)	
**Cyst types**	Microcystic	19 (73%)	7 (27%)	0.12
	Macrocytic/oligocystic	7 (100%)	0	
**von Hippel-Lindau syndrome**	Yes	4 (67%)	2 (33%)	0.42
	No	22 (81%)	5 (19%)

### Pax8 immunoreactivity in metastatic clear cell RCC involving pancreas

In nine cases of metastatic clear cell RCC involving pancreas, the proportion of Pax8 immunoreactive cells was much higher, ranging from 50 to 90%, with an average of 76% (Table 2).

### Pax8 positivity in pancreatic SCA and metastatic clear cell RCC involving pancreas

Using a 5% immunoreactivity cutoff, Pax8 positivity for pancreatic SCA was interpreted as negative for all pancreatic SCA specimens, including 23 surgical resection and 10 cytology specimens (Table 4). In contrast, Pax8 positivity in all nine cytology cases of metastatic clear cell RCC involving pancreas were interpreted as positive (Table 4). In addition, CAIX immunostain results were available in all cases of metastatic clear cell RCC involving pancreas, CD10 immunostain results were available in four such cases, and RCC immunostain result was available in one case; all these abovementioned immunostains were interpretated as positive.

**Table 4:** Percentage of Pax8 positive cases (proportion ≥ 5%)

Pancreatic serous cystadenoma, surgical specimen (*n* = 23)	0% (0/23)
Pancreatic serous cystadenoma, cytology specimen (*n* = 10)	0% (0/10)
Metastatic clear cell renal cell carcinoma involving pancreas, cytology specimen (*n* = 9)	100% (9/9)

## Discussion

In this study, we showed that Pax8 expression in pancreatic SCA is practically absent, which is consistent with a previous small-scale study [8]. Among the 23 pancreatic SCA resection specimens, only six cases showed 1% immunoreactivity, and one case showed 2% immunoreactivity, whereas ten pancreatic SCA cytology specimens did not show any Pax8 immunoreactivity. Using a 5% immunoreactivity cutoff, all cases of pancreatic SCA are interpreted as negative for Pax8 immunostains while all cases of metastatic clear cell RCC involving pancreas are interpreted as positive for Pax8 immunostains. These results indicated that Pax8 immunohistochemistry staining is a very useful adjunct in differentiating pancreatic SCA from clear cell RCC in clinical practice.

However, our results indicate three potential diagnostic pitfalls when interpreting Pax8 immunoreactivity in pancreatic SCA. First, although rare, Pax8 can be expressed in pancreatic SCA. Furthermore, Pax8 immunoreactivity was focally prominent (Fig. 1C). Therefore, caution should be exercised when interpreting small or paucicellular cytological specimens. We observed that the lowest Pax8 immunoreactivity in our cases of clear cell RCC cytology specimens was 50%, whereas Pax8 immunoreactivity was only 1% or 2% in about one fifths of our pancreatic SCA cases; thus, 5% can be considered an ideal cutoff threshold to distinguish between these two entities. Secondly, Pax8 immunoreactivity can be observed in islet cells, and sometimes such Pax8 immunoreactive islet cells can be quite close to the cyst luminal surface, causing a diagnostic pitfall (Fig. 3A and B). In such situations, additional hematoxylin and eosin deeper sections may be helpful to determine if the Pax8 immunoreactive cells are truly cyst luminal cells or adjacent islet cells. Similarly, Pax8 positivity can be seen in pancreatic well-differentiated neuroendocrine tumors with overlapping cytomorphological features [21]. If necessary, immunohistochemical staining for neuroendocrine markers such as synaptophysin or chromogranin A can be performed to further classify these Pax8 immunoreactive cells/tumors [22]. Third, along a similar line, Pax8 immunoreactivity can also be observed in lymphoid cells from adjacent lymphoid aggregates (Fig. 3C and D). Such lymphoid aggregates can be pushed towards the edge of the pancreatic tissue in pancreatic SCA, and close to the luminal surface of the cyst in some cases (Fig. 1G to I). Additional hematoxylin and eosin deeper sections will be helpful to determine if the Pax8 immunoreactive cells are truly cyst luminal cells or lymphoid aggregates. Immunohistochemical staining for lymphoid cell markers, such as CD45 (LCA), CD3, and CD20, can be performed to classify the cellular nature of Pax8 immunoreactive cells [23].

The current study has several limitations. First, all of our cases, which had already been finalized as pancreatic SCA, were retrospectively submitted for immunostaining; therefore, the tissue morphology was classic and convincing. Second, the number of cytology cases included in our study was small, owing to the relatively low prevalence of pancreatic SCA. Finally, all cases came from two closely related hospitals; thus, patient population selection bias cannot be excluded.

To conclude, our study shows that low Pax8 immunoreactivity (< 5%) can be occasionally seen in pancreatic SCA but is observed in surrounding islet cells or lymphoid aggregates. Using a 5% immunoreactivity cutoff, all cases of pancreatic SCA are interpreted as negative for Pax8 immunostains while all cases of metastatic clear cell RCC involving pancreas are interpreted as positive for Pax8 immunostains. These results indicated Pax8 immunostaining can be a useful ancillary study to distinguish pancreatic SCA from clear cell RCC in pathology specimens, both cytology and surgical specimens. To the best of our knowledge, this is the first large-scale study on Pax8 expression in surgical resection and cytology pancreatic SCA specimens.

## Data Availability

The datasets used and/or analyzed during the current study are available from the corresponding author on reasonable request.

## Abbreviations

CAIX: Carbonic anhydrase 9
CEA: Carcinoembryonic antigen
CT: Computerized tomography
EUS: Endoscopic ultrasonography
EUS-FNA: Endoscopic ultrasound-guided fine-needle aspiration
H&E: Hematoxylin and eosin
ISUP: International Society of Urologic Pathology
Pax8: Paired box 8 protein
RCC: Clear cell renal cell carcinoma
SCA: Pancreatic serous cystadenoma
VHL: Von Hippel-Lindau
WHO: World Health Organization
